# Associations Between Smoking, Stress, Quality of Life, and Oral Health Among Dental Students in Romania: A Cross-Sectional Study

**DOI:** 10.3390/medicina61081394

**Published:** 2025-08-01

**Authors:** Adina Oana Armencia, Andrei Nicolau, Irina Bamboi, Bianca Toader, Anca Rapis, Tinela Panaite, Daniela Argatu, Carina Balcos

**Affiliations:** 1 Department of Surgery, Faculty of Dental Medicine, “Grigore T. Popa” University of Medicine and Pharmacy, 700115 Iasi, Romania; adina.armencia@umfiasi.ro (A.O.A.); nicolau.andrei@umfiasi.ro (A.N.); irina.bamboi@umfiasi.ro (I.B.); bianca_toader@umfiasi.ro (B.T.); carina.balcos@umfiasi.ro (C.B.); 2 Department of Implantology, Removable Prosthesis, Denture Technology, Faculty of Dental Medicine, “Grigore T. Popa” University of Medicine and Pharmacy, 700115 Iasi, Romania; anca.stupu@umfiasi.ro; 3 Private Practice, 700106 Iasi, Romania; argatu.daniela@gmail.com

**Keywords:** smoking, dental health, stress, quality of life

## Abstract

Students, particularly those in the medical field, are exposed to various stressors that can affect their health-related behaviors, including smoking habits, with implications for oral health and quality of life. *Background and Objectives*: to analyze the relationship between smoking, oral health, perceived stress level, and self-assessed quality of life in a sample of dental students. *Materials and Methods*: The cross-sectional study included 338 students, who completed validated questionnaires and were clinically examined. Lifestyle was assessed using a smoking behavior questionnaire, stress levels were measured with the Student Stress Inventory (SSI), and quality of life was evaluated using the EQ-5D-5L instrument. The DMFT index was calculated to determine oral health status. *Results*: Among the 338 participating students, 53.8% were smokers. The lifestyle analysis revealed slightly higher average scores among non-smokers across all domains—social (3.26 vs. 3.09), attitudinal (2.75 vs. 2.97), and behavioral (3.82 vs. 3.49), but without statistically significant differences (*p* > 0.25). The mean DMFT score was 12.48, with no significant differences between smokers and non-smokers (*p* = 0.554). The SSI total score averaged 83.15, indicating a moderate level of perceived stress, again with no statistically significant differences between the groups (*p* > 0.05). However, slightly higher average stress scores among smokers may suggest the use of smoking as a coping mechanism. In contrast, quality of life as measured by EQ-5D-5L showed significantly worse outcomes for smokers across all five dimensions, including mobility (78.6% vs. 95.5%, *p* = 0.000) and self-care (93.4% vs. 100%, *p* = 0.001). Multivariable logistic regression identified smoking (OR = 1.935; *p* = 0.047) and moderate stress levels (OR = 0.258; *p* < 0.001) as independent predictors of oral health status. *Conclusions*: The results obtained suggest that smoking may function as a stress management strategy among students, supporting the relevance of integrating specific psychobehavioral interventions that address stress reduction and oral health promotion among student populations.

## 1. Introduction

The lifestyle of young people, especially students, is often characterized by unhealthy behaviors that may be associated with poorer physical and mental health outcomes. In this context, stress plays a significant role, with both positive and negative effects on health [1,2]. While it can sometimes be perceived as a motivator, it often becomes a source of maladaptation, impairing psychological balance [3].

Reactions to stress vary based on personality and individual perception of stressors, influenced by the perceived ability to cope with challenges [4,5]. Academic life is often associated with increased stress levels among students, particularly during examination periods. Studies show that stressors evolve during the years of study, with test anxiety peaking in the fifth year and low self-confidence in the third [6]. Students with better academic results tend to report lower stress levels, indicating a potential association between academic performance, lower perceived stress levels, and greater self-confidence [6].

Given the considerable association between stress and the emotional and psychological health of young people, it is important to investigate how it may contribute to the development or perpetuation of risky behaviors, such as smoking or alcohol consumption. These unhealthy behaviors are the leading cause of death worldwide, with estimates suggesting that up to one billion people could die in the 21st century if appropriate control measures are not implemented [7]. In addition, there is solid documentation that shows that smoking is a major risk factor in the development of serious chronic diseases, including various types of cancer, lung changes, and cardiovascular problems [8,9].

From a biopsychosocial perspective, perceived stress is a psychological factor that can influence health-related behaviors such as smoking, which is often adopted as a coping mechanism. In turn, smoking has well-documented detrimental effects on oral health, through both inflammatory responses and alterations in the oral microbiota. High levels of perceived stress have also been associated with increased risk of dental caries and elevated DMFT scores. For instance, Ben Hassan et al. reported a significant correlation between stress levels and DMFT (r = 0.41, *p* < 0.01) among medical students [10]. These findings support the inclusion of perceived stress within the same analytical framework as smoking and oral health, allowing for the exploration of both direct and mediated relationships. This integrative model is therefore justified in highlighting the psychobehavioral mechanisms that may influence students’ oral health status [11,12,13].

In addition to these systemic implications, smoking is significantly associated with poorer oral health outcomes. Research has shown a significant association between smoking and an increased risk of oral disease [14,15], a higher prevalence of periodontal disease, dental caries, and leukoplakia [16,17], as well as an increased frequency of oral squamous cell carcinoma [18,19]. Tumors located in the oral cavity or esophagus are much more common, ranking sixth globally in terms of incidence, both among men and women [20,21].

Medical students, in particular, represent a vulnerable group exposed to various specific stressors—educational, social, and professional—that can negatively influence health-related behaviors. Research indicates that they frequently resort to maladaptive coping strategies, such as smoking, in response to academic stress [22]. Additionally, the transition to adulthood involves making critical lifestyle decisions, which justifies the focus on evaluating oral health within this population. Perceived stress may trigger the initiation or continuation of smoking as a coping mechanism, while smoking adversely affects oral health by altering salivary composition, disrupting the oral microbiota, and exacerbating gingival inflammation. Stress can also impair oral health directly, through poor oral hygiene and unhealthy dietary choices [23]. Supporting this association, a cross-sectional study conducted in Saudi Arabia on a sample of 558 patients revealed a significant correlation between psychological distress (measured using the GHQ-12) and unfavorable oral health indicators, such as higher DMFT scores, elevated plaque and gingival indices, and poorer oral hygiene, even after adjusting for confounding variables like age, income, and smoking status [22]. These findings support the existence of both direct and indirect mechanisms through which perceived stress contributes to deteriorating oral health. Many students perceive smoking as a coping mechanism for stress, but it compromises both general and oral health, which may affect self-perceived oral health—a key component of oral health-related quality of life (OHRQoL) [24,25,26,27,28]. Despite its relevance, data on the link between smoking and OHRQoL remain limited [29,30].

Alcohol habits also form early and persist into student life, with long-term health risks [19,24]. Even though recent trends show declining use [31,32,33], alcohol remains prevalent among students.

In light of the shift toward a holistic health perspective, quality of life has become a critical health outcome indicator [34]. OHRQoL reflects how oral conditions affect daily function, communication, social life, and well-being [29,35].

The evolution of the medical paradigm towards a more holistic view, focused on social behaviours and subjective experiences of patients, has imposed the need to develop instruments that assess perceptions, emotions, and behaviors related to health. Thus, quality of life. Many students perceive smoking as a coping mechanism for stress, but it compromises both general and oral health, which may affect self-perceived oral health, a key component of oral health-related quality of life (OHRQoL), and an essential indicator for the analysis of health outcomes [34].

Oral health-related quality of life (OHRQoL) illustrates how oral problems affect individuals’ perceptions of their functioning, social integration, and mental well-being. This concept includes a variety of factors that may be associated with changes in sensory perceptions and daily habits [29,35]. The comparative importance of quality-of-life dimensions fluctuates depending on factors such as age, gender, and sociocultural norms [24,25].

The study thus aims to evaluate the relationships between smoking behavior (as an adaptive behavior) and oral health, perceived stress level, and quality of life. We started from the hypothesis that there is a significant association between smoking behavior and deterioration of oral health (assessed by DMFT), higher levels of perceived stress (measured by SSI), and lower quality of life (measured by EQ-5D-5L), with smoking being a behavioral factor that may be associated with variations in these outcomes.

## 2. Materials and Methods

### 2.1. Study Design and Study Group

The descriptive cross-sectional study was conducted between March 2025 and May 2025, in accordance with the Declaration of Helsinki and was approved by the Ethics Committee of the “Grigore T. Popa” University of Medicine and Pharmacy in Iași, Romania (No. 572/20 March 2025).

The study was conducted on a group of third- and fifth-year students from the Faculty of Dental Medicine at the “Grigore T. Popa” University of Medicine and Pharmacy in Iași. The sample size was initially calculated using a standard formula for proportions in a finite population, considering a 95% confidence level (z = 1.96), a 5% margin of error, and an estimated proportion of 0.5 [36]. This calculation indicated a minimum of 243 participants to ensure appropriate statistical power. In practice, a convenience sampling strategy was used. All eligible students (*n* = 653) were invited to participate. Participation was entirely voluntary, and 338 students who met the inclusion criteria and provided informed consent were included in the study. No random selection or stratification techniques were employed during recruitment. This method ensured compliance with ethical principles. Although a formal power analysis was not conducted, the final sample size exceeded the calculated minimum, supporting sufficient statistical power for the analyses performed. The study design also ensured participant confidentiality and anonymity, with informed consent obtained from all participants.

The inclusion criteria in the study were: age between 18 and 30 years, active students, willing to complete the questionnaires, students who signed the informed consent, who have the ability to understand and correctly answer the questionnaires, as well as subjects who are willing to participate in the assessments included in the protocol regarding oral health.

Exclusion criteria from the study were: incomplete or illogically completed questionnaires, medications affecting oral health or mental well-being, refusal to sign the informed consent form, and simultaneous participation in another study involving similar variables (to avoid bias).

### 2.2. Instruments

To assess lifestyle, oral health, perceived stress level, and quality of life, the questionnaire method was used. Specifically, smoking behavior was assessed using the questionnaire implemented in the Oral and Community Health discipline, and oral health was assessed using the DMFT index. To assess the perceived stress level, the Student Stress Inventory SSI index was used, and quality of life was measured using the EQ-5D-5L.

#### 2.2.1. Lifestyle Questionnaire

The questionnaire used in this study consists of 18 items grouped into three key domains: social, attitudes and beliefs, and behavioral, each corresponding to a core thematic question that integrates multiple sub-items for targeted statistical analysis [37]. The questionnaire used to assess lifestyle was developed and introduced in the Oral and Community Health discipline at the Faculty of Dental Medicine, “Grigore T. Popa” University of Medicine and Pharmacy, Iași, Romania, with the aim of capturing specific behaviors related to smoking, physical activity, alcohol consumption, diet, leisure activities, and other factors relevant to the student population. The instrument was validated through a pilot test conducted on a sample of 150 students, and its internal consistency, estimated by Cronbach’s α coefficient, was 0.878, indicating very good reliability [37]. In total, the questionnaire comprises 40 items, grouped into three major domains: the social domain, the attitudes and beliefs domain, and the behavioral domain. These domains allow for a comprehensive assessment of students’ lifestyles, including their perceptions, habits, and behaviors related to smoking and oral health.

The social domain: which brings together questions Q1, Q5.8, Q5.13, Q5.15, Q5.16, Q5.17, Q5.18, Q6, Q8, Q9, Q18 in the main question: “To what extent do you believe that smoking-related behaviors are influenced by social factors, such as the perceptions of others, integration into a group, social pressure and norms associated with smoking?” This question encompasses all social factors into a unified entity, and the answers given reflect the degree to which the subject perceives the impact of the social context on his or her smoking habits. Therefore, questions regarding social perceptions (e.g., “smoking as a student trait”, “smoking as a trend”, “membership of groups through smoking”) are included in the response variables, facilitating a precise statistical analysis. The set of questions that belong to this domain consists of the following items:

Q1. Do you think that being a smoker is a characteristic of a student?

Q5.8. Smoking makes you stand out.

Q5.13. Smoking eliminates shyness.

Q5.15. Smoking is a state of being with friends.

Q5.16. Smoking is triggered by curiosity and peer influence.

Q5.17. Smoking helps with fitting into the group.

Q5.18. Smoking is a fashion.

Q6. Important people to me expect me to quit smoking.

Q8. Who do you think could convince you to quit smoking?

Q9. Most important people to you believe that…

Q18. Please answer the following questions by marking with an X the answer YES or NO according to your opinion.

The attitudes and beliefs domain, which includes questions Q3, Q4, Q5, Q12, Q14, Q7, Q11, in the main question: “To what extent do you think that your personal perceptions about smoking, beliefs about this behavior, and confidence in your ability to quit smoking influence your decisions about smoking?” This question encompasses all sub-questions related to attitudes and beliefs, including perceptions about the relevance of quitting smoking, self-efficacy to quit, and personal beliefs about the effects of smoking. The responses will illustrate how each of these factors affects behaviors associated with smoking. The set of questions included in this domain comprises the following items:

Q3. Which of the following smoking-related actions do you consider most important?

Q4. For you, quitting smoking is:

Q11. How likely or unlikely is it that, over the next year, you will try to smoke only at special events?

Q12. It depends only on you to quit smoking:

Q14. If you want to quit smoking, you will succeed:

Q7. To what extent do you feel you can control yourself to:

The behavioral domain that brings together questions Q2, Q10, Q13, Q15, Q16, Q17, Q5.35, Q5.36 in the main question: “To what extent are your smoking-related behaviours, such as smoking frequency, intentions to quit and associated habits, influenced by your lifestyle and social context?” This domain addresses behavioral aspects associated with smoking and alcohol consumption frequency, desire to change, and lifestyle (e.g., alcohol consumption, participation in social activities, recreational activities, etc. The set of questions included in this domain consists of the following items:

Q2. In which of the following options do you think you fit?

Q10. How likely or unlikely is it that in the coming year you will try to quit smoking completely?

Q11. How likely or unlikely is it that in the next year you will try to smoke only at special events?

Q15. In general, how often…

Q16. How often do you use the services offered by the faculty library or others?

Q17. How often do you drink alcoholic beverages?

Q5.35. I smoke out of habit.

Q5.36. When I am working, I want to smoke.

Response options are coded from 1 to 5, where 1 means “very much”, 2 “a lot”, 3 “moderately”, 4 “a little”, and 5 “not at all” [31].

#### 2.2.2. DMFT Index

To assess oral health status, the DMFT index (Decayed, Missing, and Filled Teeth) was used. The application of this coding system allows for an objective quantitative estimation of caries experience at both individual and population levels and is recognized internationally as a standard epidemiological indicator [38,39]. The clinical examination methodology applied to the students was aligned with the World Health Organization (WHO) standards for the assessment of carious lesions. Examinations were conducted in the dental offices of the faculty, using disposable examination kits and following the clinical protocol recommended in international epidemiological studies. For each participant, an individual evaluation sheet was completed, recording the DMFT index based on the clinical status of each tooth. Dental caries was diagnosed according to strict clinical criteria established by the WHO (2013), taking into account only clearly visible cavitated lesions [38]. Each permanent tooth was evaluated individually and classified into one of three standardized categories: decayed (D), missing due to caries (M), or filled (F) [38,39]. The examinations were performed by two previously trained evaluators. To ensure the consistency and validity of the results, a calibration process was conducted before data collection. The inter-examiner calibration, assessed using the Kappa coefficient based on diagnostic agreement for a shared sample of participants, was 0.83, indicating an almost perfect level of agreement

#### 2.2.3. Student Stress Inventory (SSI) Indicator

To determine the level of stress experienced by students, the Student Stress Inventory (SSI) indicator was used. This indicator was developed with the aim of assessing the degree of stress of students in higher education institutions. It consists of 40 questions, grouped into 4 scales (each scale containing 10 questions) [40,41]:

- Subscale 1 (physical stress) addresses the impact of stress on the body from a physical perspective.
- Subscale 2 (stress in interpersonal relationships) describes how stress influences the ability to have healthy and effective interpersonal relationships.
- Subscale 3 (academic stress) focuses on the stress felt by students due to the desire to acquire advanced academic knowledge and skills in an often insufficient amount of time. This represents a key element of academic stress among students.
- Subscale 4 (environmental stress) examines how the physical and social environment affects an individual’s perceived stress, including their response to external stressors. Environmental stress highlights how the external environment influences the individual self, as well as how a person responds to stressful situations generated by external influences [40,41].

Each item in the SSI questionnaire is rated on a 4-point Likert scale: “Never” (1), “Occasionally” (2), “Frequently” (3), “Always” (4), reflecting the perceived frequency or intensity of the symptoms or situations related to stress. The scores obtained for each item are then summed to calculate: the scores for each subscale (Physical Stress, Stress in Interpersonal Relationships, Academic Stress, Environmental Stress), as well as the total SSI score that reflects the overall level of stress perceived by the student (low between 40–80, moderate between 81–121, severe between 122–160) [40,42].

This questionnaire was validated for the Romanian population, demonstrating excellent internal reliability. The Cronbach’s alpha coefficient, used to assess the internal consistency of this indicator, ranged from 0.759 to 0.886 across the 40 items, with all values falling within the range of excellent reliability [43]. The instrument is widely recognized for its utility in academic research on perceived stress among university students.

#### 2.2.4. EQ-5D-5L Indicator

The EQ-5D-5L indicator was used to assess quality of life. The EQ-5D instrument group was introduced to present and measure health-related quality of life (HRQoL) in various conditions, situations, and population groups [44]. The EQ-5D-5L was created to increase the sensitivity of previous versions and to offer participants a greater variety of options in describing their health status. The EQ-5D questionnaire creates a descriptive profile of health, with five response levels for each dimension (mobility, self-care, usual activities, pain/discomfort, and anxiety/depression). Each dimension is rated across five levels of severity, from 1 (no problems) to 5 (extreme problems), producing a five-digit health profile. Responses are converted into a single summary index score using the official EQ-5D-5L valuation set applicable to the study population. This results in a 5-digit number that describes the patient’s health status [44]. The final EQ-5D-5L score for Romania is determined by using a set of validated national values [44,45], which encode each severity level of the five dimensions of the questionnaire. Each health profile is transformed into a numerical score ranging from −0.323 (condition considered worse than death) to 1 (ideal health) [45,46]. The EQ-5D-5L instrument has been validated for the Romanian population and has been used in a series of national studies that enabled the development of a country-specific value set, as well as reference population norms [45,46].

The normality of the data distribution was checked using the Kolmogorov–Smirnov test (applying the Lilliefors correction), indicated for groups exceeding 100 participants. All questionnaires used were validated for the Romanian population.

Missing data were handled through listwise deletion, as the proportion of incomplete responses was below 5%. Potential confounding variables such as socioeconomic status and alcohol consumption were not included in the analysis due to the limited scope of the questionnaire, and this is acknowledged as a limitation. To reduce response bias, participation was anonymous and voluntary, and the questionnaires were completed without time constraints, in a non-evaluative environment.

### 2.3. Statistical Analysis

SPSS 26.0 software (IBM, Armonk, NY, USA) was used for statistical analysis of the data. A descriptive analysis was performed to determine the frequencies and percentages of demographic variables. The *t*-test and Mann–Whitney U-test were used to compare scores between groups (smokers vs. non-smokers). The Chi-square (χ^2^) test was applied for differences between proportions. ANOVA analysis was used to evaluate the variation of scores according to a parameter, and the Pearson correlation coefficient was applied to explore the relationships between quantitative variables. Multiple logistic regression was applied to identify independent variables associated with oral health, as measured by the DMFT score. The statistical significance of the results was established by means of the *p*-value, using a threshold of 0.05 to determine the relevance of the observed relationships.

## 3. Results

### 3.1. Socio-Demographics

The socio-demographic distribution of participants shows a predominance of women (74.6%) over men (25.4%), which may reflect their greater presence in the targeted field or a greater willingness to participate in research. In terms of age, the majority of respondents fall within the 21–24 age range (58.9%), followed by those over 25 (39.1%), while young people between 18–20 are poorly represented (2.1%). In terms of year of study, participants in the 5th year constitute 79.6% of the sample, compared to 20.4% in the 3rd year, suggesting a greater involvement of students in older years. Also, 61.2% of respondents come from urban areas, compared to 38.8% from rural areas, which may influence the attitudes and behaviors analyzed in the study. 53.8% of the participants are smokers (Table 1).

### 3.2. Lifestyle and Smoking Behaviour

The results of the study showed that 50.4% of women and 64.0% of men were smokers. For social influences, the mean score was 3.09 ± 1.65 among smokers and 3.26 ± 1.76 among non-smokers (F = 4.40, *p* = 0.29). Regarding attitudes, the scores were 2.96 ± 2.64 vs. 2.74 ± 1.01 (F = 5.75, *p* = 0.51), and for the behavioral dimension, 3.49 ± 6.16 vs. 3.82 ± 8.84 (F = 3.44, *p* = 0.25). The lifestyle analysis did not reveal statistically significant differences between smokers and non-smokers in terms of social influences, attitudes, and beliefs related to smoking, and behaviors associated with it (*p* > 0.05) (Table 2).

These results may reflect the presence of common social norms or a high level of similar informational exposure, regardless of consumption behavior. Thus, prevention or behavior change interventions should consider not only smoking status but also the contextual and psychosocial factors that shape representations and decisions related to smoking among young people.

### 3.3. Oral Health (DMFT Index)

**Figure 1:**
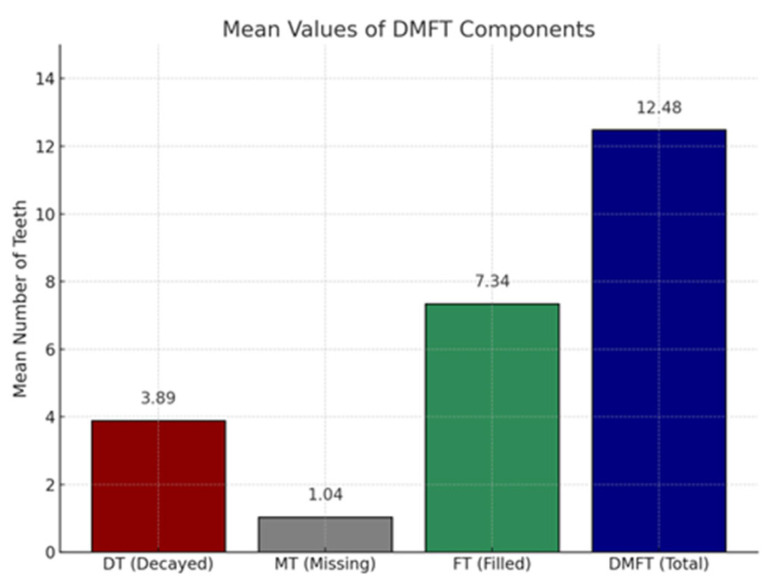
Average score of the DMFT index. Each color represents a component of DMFT.

The assessment of dental health status using the DMFT index revealed an average score of 12.48 (M = 24.75, SD = 12.12), indicating a high level of dental caries experience. The FT component has the highest value (7.34), indicating increased access to dental services, especially for restorative treatments. In contrast, the DT component has an average value of 3.89, which highlights a fairly high frequency of carious lesions. The MT component obtained the lowest score (1.04) (Figure 1).

### 3.4. Perceived Stress (SSI)

The application of the Student Stress Inventory (SSI) questionnaire on the studied group revealed an average value of 21.85 for the physical dimension (D1), which signifies a level of moderate stress (19–29). The interpersonal relationships dimension (D2), with an average value of 19.01, is at the lower limit of moderate stress, indicating that the participants’ social relationships are generally effective, but can become fragile in situations of psycho-emotional overload.

Regarding the academic dimension (D3), the average score of 21.77 suggests the presence of moderate difficulties related to motivation, time management, and efficiency in carrying out educational activities.

**Figure 2:**
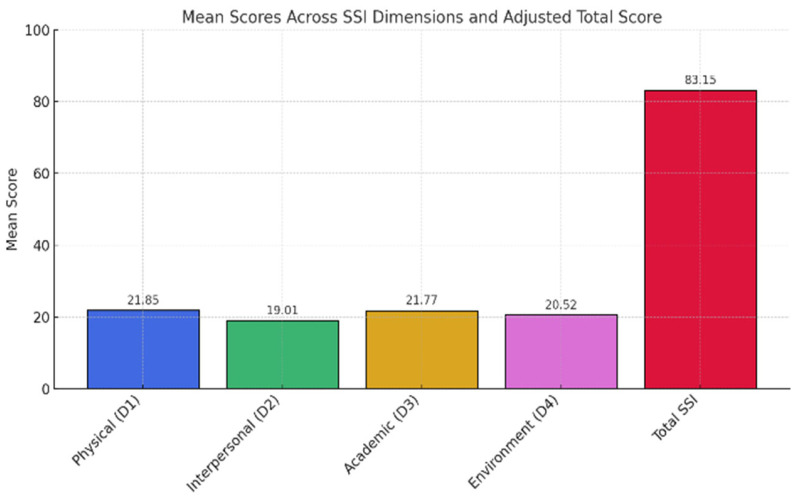
Distribution of mean scores and total scores on SSI dimensions. Each color represents a different dimension of SSI indicator.

The social dimension (D4), with an average score of 20.52, also falls within the moderate stress area, indicating a partial balance between the academic and social environments. The final score obtained is 83.15, a value that corresponds to moderate perceived stress, pointing to a general condition marked by recurrent adaptive difficulties, without exceeding the level of severe dysfunction, but indicating an increased psychological vulnerability to academic, interpersonal, and contextual stressors (Figure 2).

The comparative analysis of Student Stress Inventory (SSI) scores revealed no statistically significant differences between smokers and non-smokers across the four domains: physical stress (21.91 ± 4.72 vs. 21.77 ± 4.49; F = 0.073, *p* = 0.787), interpersonal relationship stress (18.73 ± 4.89 vs. 19.33 ± 4.66; F = 1.357, *p* = 0.245), academic stress (22.00 ± 4.51 vs. 21.48 ± 5.18; F = 0.964, *p* = 0.327), and environmental stress (20.78 ± 5.78 vs. 20.20 ± 5.50; F = 0.885, *p* = 0.348). Although not statistically significant (all *p* > 0.05), the slightly higher scores among smokers suggest a trend toward higher perceived stress, possibly indicating the use of smoking as a coping strategy (Table 3).

### 3.5. Quality of Life (EQ-5D-5L)

**Figure 3:**
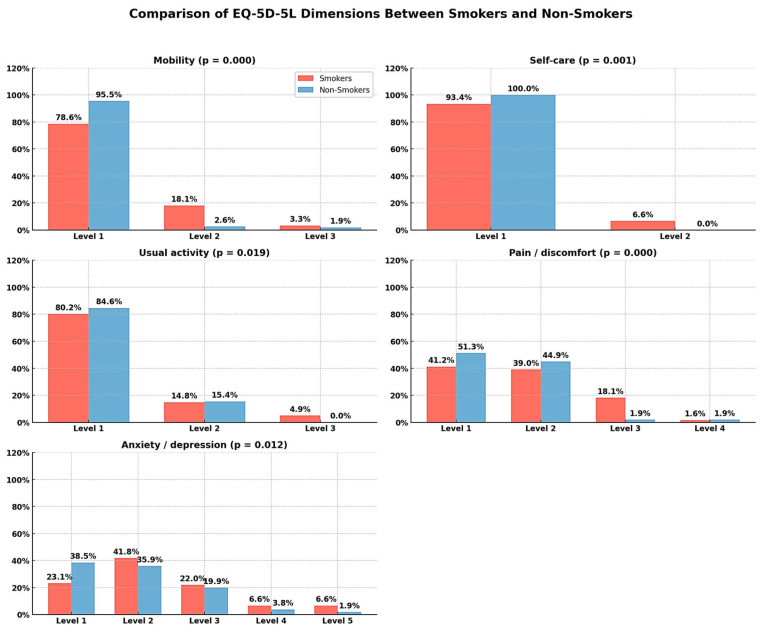
Distribution of EQ-5D-5L scores obtained using the validated set of values for Romania.

Regarding the quality of life assessed by the EQ-5D-5L instrument, using the set of values validated for Romania, it is observed that most of the scores are in the range of 0.80–1.00, which suggests a good to excellent general health status for most of the participants (Figure 3). The significant point reached at the maximum score (1000) indicates a large number of respondents who recorded the absence of changes for all dimensions of the quality of life assessed. Also, a moderate presence of scores between 0.60 and 0.80 is noted, which indicates the presence of mild to moderate problems in a small part of the study group. Regarding the quality of life assessed by the EQ-5D-5L instrument, using the set of values validated for Romania, it is observed that most of the scores are in the range of 0.80–1.00, which suggests a good to excellent general health status for most of the participants (Figure 3). The significant point reached at the maximum score (1000) indicates a large number of respondents who recorded the absence of changes for all dimensions of the quality of life assessed. Also, a moderate presence of scores between 0.60 and 0.80 is noted, which indicates the presence of mild to moderate problems in a small part of the study group.

The comparative analysis of the dimensions of quality of life measured by the EQ-5D-5L questionnaire revealed statistically significant differences between smokers and non-smokers in all five domains assessed. In the mobility domain, a significantly lower percentage of smokers (78.6%) reported no difficulties (Level 1), compared to 95.5% of non-smokers (χ^2^, *p* = 0.000), indicating more frequent mobility impairment among smokers. Also, in the self-care domain, all non-smokers declared the absence of difficulties, compared to 93.4% of smokers, a difference also significant (χ^2^ = 0.001).

Usual activities were more frequently affected in smokers (19.7% Level 2 and 3) than in non-smokers (15.4%), a statistically significant difference (χ^2^ = 0.019). The largest disparities were observed in the pain/discomfort dimension, where 58.8% of smokers reported various forms of discomfort (Level 2–4), compared to 48.7% of non-smokers (χ^2^ = 0.000). Regarding anxiety/depression, smokers reported higher levels of psychological impairment (Level 3–5: 35.2%) compared to non-smokers (25.6%), the difference being significant (χ^2^ = 0.012). These results suggest a more pronounced deterioration in quality of life among smokers, with an impact on both physical functionality and mental state and perceived discomfort (Table 4).

Table 5 presents the comparative analysis of DMFT (Decayed, Missing, and Filled Teeth) scores according to smoking status. Although the mean DMFT score was slightly lower among smokers (M = 24.39, SD = 11.74) than among non-smokers (M = 25.17, SD = 12.52), the difference was not statistically significant (F = 0.351, *p* = 0.554). These findings indicate that, within the analyzed group, smoking does not appear to have a significant impact on the DMFT index. While smoking is often linked to poor oral health outcomes, the present data do not reveal a clear distinction between smokers and non-smokers in terms of caries experience or dental treatment (Table 5).

### 3.6. Correlation Analysis

Pearson correlation analysis highlights a significant association between smoking status and several dimensions related to quality of life. Thus, smoking is negatively correlated, albeit weakly, with self-care (r = −0.178), pain or discomfort (r = −0.175), and negative emotional state (r = −0.176), indicating an association between smoking and a reduced perception of physical and mental health.

Perceived stress—particularly in its physical, academic, and environmental dimensions—was significantly correlated with several aspects of quality of life. Specifically, physical stress showed a moderate positive correlation with pain (r = 0.42, *p* < 0.01) and limitations in daily activities (r = 0.37, *p* < 0.05), suggesting that individuals experiencing higher levels of physical stress also reported greater functional impairment. Academic stress was positively correlated with reduced self-care capacity (r = 0.35, *p* < 0.05), while environmental stress was significantly associated with both increased pain perception (r = 0.40, *p* < 0.01) and difficulties in carrying out usual tasks (r = 0.38, *p* < 0.05). These findings underscore the substantial impact of stress on overall functioning and perceived quality of life.

**Figure 4:**
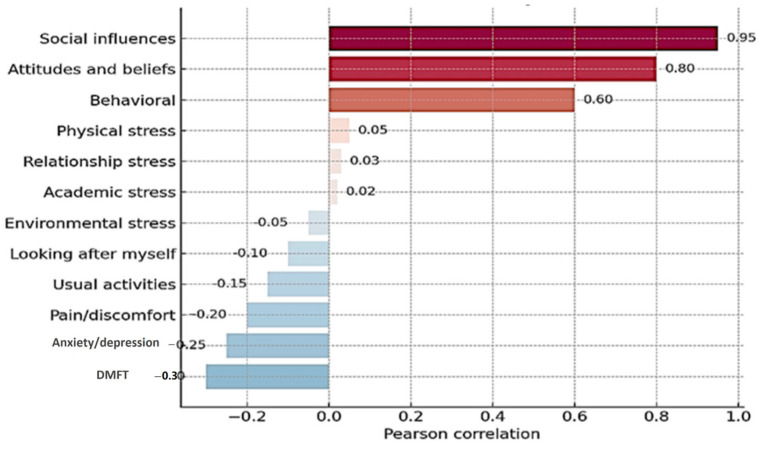
Pearson Correlation with Smoking status. The Pearson correlation between study variables and smoking behavior. Each color represents one variable for the correlation between DMFT and all three indicators.

Although the DMFT index was not significantly correlated with smoking status (*p* = 0.554), it was weak but positively associated with relational stress (r = 0.21, *p* < 0.05) and the presence of pain (r = 0.24, *p* < 0.05). This may point to indirect pathways through which psychosocial stress could affect oral health outcomes (Figure 4).

The results indicate that smoking is associated with the perception of poor general health rather than with actual stress, and stress appears to be an important determinant of self-rated quality of life.

The multivariable logistic regression analysis identified study year, moderate perceived stress, and smoking status as significant independent predictors of oral health status (Table 6). Students in the final year of study were 4.4 times more likely to present with good oral health (DMFT ≤ 10) compared to those in earlier years (OR = 4.409; 95% CI: 1.306–14.890; *p* = 0.017), suggesting that greater academic experience may be associated with improved oral health behaviors.

Additionally, students who reported moderate stress levels had a significantly lower likelihood of maintaining good oral health compared to those with low stress (OR = 0.258; 95% CI: 0.125–0.534; *p* < 0.001), supporting the role of psychological stress as a risk factor. In contrast, high stress was not significantly associated with oral health status (*p* = 0.241).

Non-smokers had significantly higher odds of good oral health compared to smokers (OR = 1.935; 95% CI: 1.010–3.706; *p* = 0.047), reaffirming the detrimental impact of smoking on oral health.

Gender, age, and place of residence were not statistically significant predictors in the fully adjusted model. Multivariable logistic regression also identified smoking as a significant independent predictor of poor oral health. Specifically, smokers were nearly twice as likely to have a higher DMFT score compared to non-smokers (OR = 1.935, 95% CI: 1.01–3.71, *p* = 0.047) (Table 7).

In contrast, moderate levels of perceived stress were inversely associated with high DMFT scores (OR = 0.258, *p* < 0.001), suggesting a potentially protective effect. These findings support the relevance of behavioral and psychological factors in influencing oral health among university students.

Although the association between smoking and higher DMFT scores was statistically significant (OR = 1.935, 95% CI: 1.01–3.71, *p* = 0.047), the clinical relevance of this effect size should be interpreted with caution. While the odds ratio suggests an increased risk, the modest confidence interval indicates that the actual impact on oral health may be moderate. Similarly, the inverse relationship between moderate stress and DMFT (OR = 0.258, *p* < 0.001) was statistically strong, but further studies are needed to confirm whether this association translates into clinically meaningful improvements in oral health behaviors or outcomes.

## 4. Discussion

Given the complexity of oral and psychosocial health, this research examined how health-related behaviors, particularly smoking, are associated with, or are a consequence of, dental health status determined by the DMFT index, perceived stress level assessed by the Student Stress Inventory (SSI), and self-assessed quality of life using the EQ-5D-5L instrument. In a young population, academic pressures and lifestyle choices may influence the three dimensions–physiological, psychological, and qualitative–which can interact in subtle yet significant ways. A behavioral factor potentially related to all three domains provides an integrated perspective on overall health, in which smoking is not merely considered an isolated risk factor, but rather a possible mediator between stress, oral health, and quality of life.

Regarding socio-demographic characteristics, a higher prevalence of male subjects was observed among smokers, an aspect that aligns with the information published by the World Health Organization [47]. Regarding the quality of life related to oral health, smokers were found to have considerably lower quality-of-life scores than non-smokers. This observation is reinforced by the conclusions presented by [28,29], which highlighted a correlation between smoking and impaired quality of life.

Existing studies have shown that, compared to active smokers, non-smokers reported significantly better quality of life scores, as well as lower levels of anxiety and depressive symptoms [48]. In most studies, the association between smoking and the psychological dimension of quality of life was more pronounced than the link between smoking and the physical dimension [48].

Similar to our findings, a study conducted in India revealed scores that showed an lower quality of life scores in quality of life among smokers [49]. Also, a study conducted in Thailand, which included 87,134 adults, demonstrated that smokers had a lower quality of life in all its dimensions [50]. Another study conducted in Turkey highlighted a significant link between vicious habits (including smoking) and a lower quality of life, compared to patients who did not have these habits [51]. The harmful effects of smoking on oral tissues can be seen as a factor that has been associated with poorer oral health and deteriorated quality of life in active smokers [30].

The DMFT index indicated a high prevalence of dental caries. Existing studies have demonstrated a link between smoking and the proliferation of cariogenic bacteria [52,53]. Nicotine may be associated with increased colonization and activity of Streptococcus mutans [54]. In addition to this, other factors such as a diet rich in fermentable carbohydrates, poor oral hygiene, genetic predisposition, and individual susceptibility also contribute to the development and progression of carious lesions [47].

Given these contributing factors, it is important to interpret DMFT data with caution, taking into account the index’s inherent limitations and the influence of potential confounding variables. The DMFT score does not differentiate between decayed, extracted, or filled teeth, nor does it indicate whether teeth were lost for reasons unrelated to caries, thereby limiting its overall relevance and interpretative accuracy [39].

Furthermore, smoking has been associated with alterations in salivary secretion by reducing its buffering capacity and by altering both the composition and flow of saliva, thus creating a favorable environment for the onset and progression of the carious process [54].

Results from the National Health and Nutrition Examination Survey (NHANES) conducted between 2011 and 2016 showed that 40–50% of adult smokers aged 20–64 years had untreated tooth decay [55]. Studies conducted in Italy [56] and Finland [57] have shown that smokers have higher DMFT (decayed, missing, filled teeth) scores than non-smokers.

The total score for perceived stress indicated a moderate level of stress. The results of the study are consistent with the data available in the literature. Restrepo et al. reported average levels of academic stress in a study conducted on a group of Colombian students [58]. However, this can lead over time to unfavorable outcomes such as dropping out of school, adopting other unhealthy behaviors (using illicit substances, smoking, or alcohol as adaptive means), and, ultimately, to the emergence of mental problems (depression, anxiety, or generalized stress) [39,59].

In general, students in their first years of study go through an initial maturation stage, in which stressors and their effects may vary, correlating with increasing personal responsibilities [60]. In addition, students frequently exhibit higher levels of stress compared to the general population [61], associated with reduced levels of self-esteem, optimism, and self-efficacy [62]. In the same vein, a recent study conducted on medical students in Germany demonstrated that the level of perceived stress, as well as the level of emotional distress, is higher than that reported among young people in the general German population [62].

Regarding quality of life, most participants obtained scores that suggest a generally favorable view of their health. Students are in a sociodemographic phase of existence in which stress-related disorders are more common. Also, the university year involves the allocation of time and financial resources to students without providing the certainty of a satisfactory result. This pressure can determine an impaired perception of the quality of life in its various aspects: health, physical condition, psychological, living environment, and social relationships [26,63,64].

In the present study, the hypothesis was only partially supported by the results. The only statistically confirmed relationship is between smoking and deterioration in quality of life. No significant associations were identified between smoking and DMFT score or perceived stress level.

The original elements of this study can be highlighted by the following aspects: multidimensional approach to the impact of smoking because the research is not limited to the analysis of smoking as a simple risk factor for oral health, but includes three separate dimensions: oral health (assessed by DMF-T), perceived stress (SSI) and quality of life (EQ-5D-5L); the focus of the study on dental students, which gives relevance to the analysis of perceptions and behaviors associated with health in an educated group and potentially influential in the formation of future opinions; the assessment of quality of life with EQ-5D-5L in relation to smoking is less common in Romanian studies regarding students’ lifestyle; direct and indirect correlation between psychosocial and clinical variables, because the research analyzes not only direct interactions, but also potential indirect interconnections between variables (for example, the relationship between stress and DMFT score), helping to elucidate the subtle mechanisms through which smoking can affect general health; last but not least, results that are contrary to initial expectations, only the quality of life being significantly affected. To minimize selection bias, all participants were recruited from the same institution using consistent inclusion and exclusion criteria. Participation was voluntary and based on informed consent. To reduce measurement bias, validated and standardized instruments (DMFT, SSI, EQ-5D-5L) were used, with prior calibration of examiners for clinical assessment.

The limitations of the study are primarily related to its cross-sectional, observational design, with data collected at a single point in time, which restricts the ability to establish causal relationships between smoking and the variables studied (DMFT, stress, quality of life).

The comparative analysis of DMFT scores between smokers and non-smokers did not reveal statistically significant differences. However, this lack of statistical significance should be interpreted with caution, given the relatively small size of the sub-groups and the absence of a formal power analysis for this specific comparison. Furthermore, in the present study, no adjustments were made for potential confounding factors involved in the etiology of dental caries, such as diet, oral hygiene, socio-economic status, or access to dental care services. Therefore, the interpretation of the relationship between caries status (DMFT) and smoking should be considered relative and constrained by the observational nature of the study. For this reason, future research should include adjusted multivariate models that allow for an independent assessment of the influence of smoking on oral health in the context of other behavioral and social determinants.

Associations can be observed; however, it cannot be stated with certainty that smoking causes the observed changes. Moreover, the variables were derived from self-reported questionnaires, which carry the risk of recall and social desirability bias. Consequently, respondents may underestimate or overestimate certain behaviors (e.g., the number of cigarettes smoked), perceived stress levels, or other conditions. Although the questionnaires used had been previously validated and demonstrated good internal consistency, some subdomains—especially those related to lifestyle—may not be fully adapted to the cultural or academic context of the group studied. This may lead to very high correlations between certain subdomains and reduce the validity of the instrument for this specific population.

In addition, the sample is not nationally representative, as participants were recruited from a single higher education institution. This limitation reduces the possibility of generalizing the results to the broader youth or student population in Romania. The gender imbalance, with a predominance of female participants, may also influence the findings, especially regarding perceptions of stress, lifestyle, and quality of life. Furthermore, the specific socio-demographic characteristics of this group could affect perceptions, behaviors, and stress levels, further restricting the extent to which these conclusions can be extrapolated to other youth populations or educational contexts.

Given these considerations, the results should be interpreted with caution, and future research should employ adjusted multivariate models to control for potential confounding factors (such as diet, oral hygiene, socioeconomic status, and access to dental care), allowing for a more rigorous independent assessment of the influence of smoking on oral health, stress, and quality of life.

Taken together, these aspects highlight the observational and self-reported nature of the research, the potential confounding factors, as well as the limitations associated with sampling. The explicit acknowledgment of these aspects ensures a more rigorous and balanced evaluation of the results and helps to avoid overestimating their broader implications.

The study did not include biological markers or clinical parameters beyond the DMFT index, which could have provided a more objective and comprehensive assessment of oral and general health.

The cross-sectional design also limits the ability to examine temporal relationships between stress, smoking, and changes in oral health behaviors over time.

Given the observational and cross-sectional nature of this study, the results should be interpreted with caution, as they do not allow for establishing direct causal relationships between smoking, stress, and oral health. Additionally, potential biases related to self-reporting and sampling may be present, and the absence of objective clinical or biological variables limits the depth of the analysis. Nevertheless, the findings may help shape intervention strategies, particularly in university settings, where integrated programs aimed at smoking prevention and stress reduction could be implemented. Such interventions may positively impact not only oral health but also the overall well-being of students. Furthermore, future research should include longitudinal studies to assess causal relationships and the evolution of associated behaviors over time, as well as interventional studies to evaluate the effectiveness of health education programs among young people.

Based on the results of this study, several relevant directions for future research can be identified. First, an in-depth investigation is warranted into how smoking may function as a coping mechanism for stress, given its observed impact on students’ quality of life, even in the absence of a statistically significant correlation with perceived stress levels. Second, longitudinal studies are needed to monitor changes over time in smoking behavior, oral health, and psychological well-being, providing a clearer perspective on causal relationships. Finally, interventional research should examine the effectiveness of integrated educational programs that include stress management, awareness of the risks associated with smoking, and the promotion of oral health, to reduce risky behaviors and contribute to improved quality of life among the student population.

## 5. Conclusions

The study revealed significant differences between smokers and non-smokers in terms of quality of life, with smokers reporting higher levels of functional, physical, and psychological impairment. The dimensions most commonly reported as impaired included mobility, self-care, usual activities, pain, and emotional state. In contrast, no significant differences were identified in terms of DMF-T score or perceived stress level, suggesting that, in the context of this sample, smoking does not directly influence oral health or stress, but appears more strongly associated with lower quality of life scores. These results highlight the importance of approaching smoking behaviour from an integrated perspective, which considers its impact on overall well-being, not just on isolated clinical or psychological indicators. Given the gender imbalance in the sample—predominantly female—future research should aim to include gender-balanced samples, as gender may influence the perception of stress, lifestyle, and quality of life.

## Figures and Tables

**Table 1:** Demographic characteristics of the study group.

Category	Variable	Frequency	Percentage (%)
Gender	Man	86	25.4
	Woman	252	74.6
Age	18–20 years old	7	2.1
	21–24 years old	199	58.9
	Over 25 years old	132	39.1
Year of study	Year III	69	20.4
	Year V	269	79.6
Residence environment	Urban	207	61.2
	Rural	131	38.8
Smoker	Yes	182	53.8
	No	156	46.2

**Table 2:** Average results of the lifestyle questionnaire domains.

Field	Representative Question	Smoker		F	*p*
		Yes (Mean Value + DS)	No (Mean Value + DS)		
Social (Social influences, social pressure, social integration)	“To what extent do you think smoking behaviours are influenced by social factors, such as the perceptions of others, integration into a group, social pressure, and norms associated with smoking?”	3.09 ± 1.65	3.26 ± 1.76	4.40	0.29
Atittudes (Perceptions about smoking, beliefs, self-efficacy)	“To what extent do you think your personal perceptions about smoking, beliefs about this behaviour, and confidence in your ability to quit smoking influence your smoking decisions?”	2.96 ± 2.64	2.74 ± 1.01	5.75	0.51
Behavioural (Actual behaviour, behavioural intentions, lifestyle)	“To what extent are your smoking-related behaviours, such as smoking frequency, intentions to quit, and associated habits, influenced by your lifestyle and the social context in which you find yourself?”	3.49 ± 6.16	3.82 ± 8.84	3.44	0.25

**Table 3:** Results of the analysis of the Student Stress Inventory (SSI) questionnaire data, comparing smokers and non-smokers.

Field	Smoker		F	*p*
	Yes (Mean Value + DS)	No (Mean Value + DS)		
Physical stress	21.91 ± 4.72	21.77 ± 4.49	0.073	0.787
Interpersonal Relationship Stress	18.73 ± 4.89	19.33 ± 4.66	1.357	0.245
Academic Stress	22.00 ± 4.51	21.48 ± 5.18	0.964	0.327
Environmental Stress	20.78 ± 5.78	20.20 ± 5.50	0.885	0.348

**Table 4:** Results of data analysis of the EQ-5D-5L questionnaire, comparing smokers and non-smokers.

Field	Level (1–5)	Smoker		χ^2^
		Yes (%)	No (%)	
Mobility	Level 1	78.6	95.5	0.000
	Level 2	18.1	2.6	
	Level 3	3.3	1.9	
Self-care	Level 1	93.4	100	0.001
	Level 2	6.6		
Usual activity	Level 1	80.2	84.6 15.4	0.019
	Level 2	14.8		
	Level 3	4.9		
Pain/discomfort	Level 1	41.2	51.3	0.000
	Level 2	39.0	44.9	
	Level 3	18.1	1.9	
	Level 4	1.6	1.9	
Anxiety/depression	Level 1	23.1	38.5	0.012
	Level 2	41.8	35.9	
	Level 3	22.0	19.9	
	Level 4	6.6	3.8	
	Level 5	6.6	1.9	

**Table 5:** Comparison of mean DMFT score between smokers and non-smokers.

Do You Smoke?	*n*	Mean of DMFT	Std. Deviation	F	Sig.
Yes	182	24.3901	11.74388	0.351	0.554
No	156	25.1731	12.51794		
Total	338	24.7515	12.09546		

**Table 6:** Parameter estimates from the Binary Logistic Regression predicting the likelihood of good oral health based on sociodemographic, behavioral, and psychological factors.

DMFT ^a^		B	Std. Error	Wald	df	Sig.	Exp(B)	95% Confidence Interval for Exp(B)	
								Lower Bound	Upper Bound
DMFT ≤ 10”—sănătate orală relativ bună	Intercept	−1.717	0.300	32.850	1	0.000			
	Gender	0.378	0.363	1.089	1	0.297	1.460	0.717	2.971
	Age	0.415	0.310	1.788	1	0.181	1.514	0.824	2.780
	Study year	1.484	0.621	5.709	1	0.017	4.409	1.306	14.890
	Place of residence	−0.123	0.338	0.132	1	0.717	0.885	0.456	1.717
	[High stress level]	−0.909	0.775	1.376	1	0.241	0.403	0.088	1.841
	[Moderate stress level]	−1.353	0.370	13.379	1	0.000	0.258	0.125	0.534
	[Non-Smoker]	0.660	0.332	3.960	1	0.047	1.935	1.010	3.706

^a^ The reference category is: DMFT > 10”—Poor oral health.

**Table 7:** Multivariate logistic regression analysis—predictors of oral health (DMFT score).

Independent Variable	OR (95% CI)	*p*-Value
Smoking (smoker vs. non-smoker)	1.935 (95% CI: 1.01–3.71)	0.047
Moderate stress (vs. low stress)	0.258 (CI not specified)	<0.001

## Data Availability

The data supporting the reported results can be obtained upon request in the form of datasets available in the Community Dentistry Discipline, Department of Surgery, Faculty of Dental Medicine, “Grigore T. Popa” University of Medicine and Pharmacy, Iasi, Romania.
